# Opinions of general practitioners about psychotherapy and their relationships with mental health professionals in the management of major depression: A qualitative survey

**DOI:** 10.1371/journal.pone.0190565

**Published:** 2018-01-31

**Authors:** Hélène Dumesnil, Thémis Apostolidis, Pierre Verger

**Affiliations:** 1 Aix Marseille Univ, INSERM, IRD, SESSTIM, Sciences Economiques & Sociales de la Santé & Traitement de l’Information Médicale, Marseille, France; 2 ORS PACA, Observatoire Régional de la Santé Provence-Alpes-Côte d’Azur, Marseille, France; 3 Aix Marseille Univ, LPS, Aix en Provence, France; 4 Aix Marseille Univ, IRD, AP-HM, SSA, VITROME, IHU-Méditerranée Infection, Marseille, France; Otto von Guericke University Magdeburg, GERMANY

## Abstract

**Background:**

French general practitioners (GPs) refer their patients with major depression to psychiatrists or for psychotherapy at particularly low rates.

**Objectives:**

This qualitative study aims to explore general practitioners' (GP) opinions about psychotherapy, their relationships with mental health professionals, their perceptions of their role and that of psychiatrists in treating depression, and the relations between these factors and the GPs' strategies for managing depression.

**Methods:**

In 2011, in-depth interviews based on a semi-structured interview guide were conducted with 32 GPs practicing in southeastern France. Verbatim transcripts were examined by analyzing their thematic content.

**Results:**

We identified three profiles of physicians according to their opinions and practices about treatment strategies for depression: pro-pharmacological treatment, pro-psychotherapy and those with mixed practices. Most participants considered their relationships with psychiatrists unsatisfactory, would like more and better collaboration with them and shared the same concept of management in general practice. This concept was based both on the values and principles of practice shared by GPs and on their strong differentiation of their management practices from those of psychiatrists,

**Conclusion:**

Several attitudes and values common to GPs might contribute to their low rate of referrals for psychotherapy in France: strong occupational identity, substantial variations in GPs' attitudes and practices regarding depression treatment strategies, representations sometimes unfavorable toward psychiatrists. Actions to develop a common culture and improve cooperation between GPs and psychiatrists are essential. They include systems of collaborative care and the development of interdisciplinary training common to GPs and psychiatrists practicing in the same area.

## Introduction

Although the density of psychiatrists in France is among the highest in Europe, French general practitioners (GPs) refer their patients with major depression to psychiatrists or for psychotherapy at particularly low rates [1,2]. This is especially striking in that psychotherapy is recommended as the first-line treatment for major depression of mild to moderate intensity and as a complement to pharmacological treatment for severe depression in France [3] and elsewhere [4,5].

A quantitative survey that we conducted in 2011 among a national panel of GPs identified a dual paradox in GPs' opinions and practices related to psychotherapy in the management of major depression [6,7]. First, GPs on the whole had favorable opinions about psychotherapy and recognized its effectiveness for treating depression, but they rarely suggested it to their patients with mild to moderate depression; instead they prescribed antidepressants [6]. This discrepancy between their opinions and their practices may be explained by obstacles related to access to psychotherapy (unequal distribution of mental health professionals, long wait times for psychiatrist appointments for new patients, and French policy, which does not reimburse psychotherapy by psychologists) or patient reluctance. Our analyses, however, did not find that any of these obstacles were associated with the GPs' treatment choices [6].

In their theoretical model of access to mental health care at different points along patients’ health care trajectory [8], Goldberg and Huxley argue that GPs play an important role as gatekeepers to access to psychiatric care. In particular, they point out that a principal obstacle to this access lies on the interface between primary care providers and the organizations and professionals specialized in mental health care.

Both GPs’ relationships with mental health professionals and their representations of these specialists' practices might influence the access of their patients with psychiatric disorders to adequate care. Although the literature reports numerous difficulties in relationships between GPs and psychiatrists [9–12], we found only a single qualitative study dealing with the issue of referrals for psychotherapy by GPs of their patients with depression [13]. This study of Swedish GPs found that the GPs questioned did not consider psychotherapy as a treatment in its own right for major depression and favored the use of antidepressants, regardless of the severity of depression.

In 2011, together with the quantitative survey, we conducted an exploratory qualitative survey of GPs in private practice to analyze their opinions and practices in the management of depression. This article, based on that qualitative study, seeks to understand the paradoxes described above by analyzing: 1) the opinions of these physicians about psychotherapy (objective 1); 2) their relationships with mental health professionals (objective 2), and 3) the more general way that GPs perceive their profession and their role, as well as that of psychiatrists, in the treatment of major depression (objective 3).

## Materials and methods

### Recruitment of participants

We randomly selected 50 physicians in the database of the French National Health Insurance Fund for southeastern France among GPs practicing in the city of Marseille on January 1, 2011. The random drawing was stratified by physicians' age (younger than 50 years; 50 years or older) and sex.

We sent a letter to these physicians, announcing and briefly describing this study, its objectives, and its procedures, and then contacted them by telephone, to obtain their agreement to participate and to make an appointment for the interview. Their written consent was collected at the interview. To comply with the confidentiality and anonymity of the interviews promised to the GPs, the information collected during the interviews cannot be shared in its complete form.

### Data collection

Two psychologists used a semi-structured interview guide to conduct in-depth face-to-face interviews. The instrument, drafted with a group of experts (a GP, a mental health specialist, an epidemiologist, and 2 social psychologists), and pilot-tested among 6 GPs, was intended to explore a wide range of potentially relevant issues about the management of major depression. It covered, in particular, an analysis of GPs’ choices and strategies when starting to treat patients for major depression, their relations with mental health specialists, their opinions of psychotherapy and pharmacotherapy, and their perceived role, self-efficacy, and difficulties in managing patients with major depression (S1 Appendix). Although the instrument was prepared to ensure that the same themes were studied in each interview, there were no predetermined responses, and participants were encouraged to talk freely. Data were collected from March to May 2011. Each psychologist interviewed half the GPs. The interviews lasted 36 minutes on average (15–116 minutes) and were all audiotaped with the GP’s consent, then completely transcribed manually (as Word files), and anonymized by both researchers. All the interviews were conducted, transcribed, and analyzed in French.

At the conclusion of the interviews, the participants also completed a short questionnaire about their individual (age, sex) and professional (years of practice, group or solo practice, and training in mental health) characteristics.

### Data analysis

The two social psychologists who conducted the interviews separately performed thematic analyses of all the interviews and then crossed their results (triangulation of researchers). We performed an analysis of thematic content to analyze the data related to GPs’ opinions about psychotherapy and their collaboration with psychiatrists.

The two psychologists analyzed each subject’s words according to their thematic content [14], applying a common multiple-step method for each transcript: First, they familiarized themselves with the data by repeatedly reading the transcripts and listening to the interview audiotapes. Next, an initial framework for interpretation was developed based on the study objectives and the interview guide, in the form of a grid or table for each interview (Table 1). The themes that emerged spontaneously from a participant's discourse were distinguished from those in response to questions from the interview guide, because spontaneously mentioning a theme rather than discussing it only once it is raised by the interviewer can be revealing of the importance the interviewee attributes to it. Spontaneous mention may indicate, for example, that the theme is frequently encountered, presents the most difficulties, or is a major concern or worry.

**Table 1:** Vertical analysis (by interview).

Interview n°…					
Theme	Subtheme	N° page/line	Spontaneous (S)/In response to (R)^1^	Excerpt from interview	Comments
…	…	*Page* x/line y	R	…	…
…	…	*Page* x’/line y’	I	…	…

^1^ Spontaneous (S): spontaneous mention of a theme / In response to (R): evocation of a theme in response to a question from the interviewer guide

Next, a cross-sectional analysis was performed for each theme, and a table produced for it (Table 2).

**Table 2:** Horizontal analysis (by theme).

Theme: …					
Interview	Subtheme	N° page/line	Spontaneous (S)/ In response to (R)^1^	Excerpt from interview	Comments
1	…	*Page* x/line y	R	……	…..
2					
3	…	*Page* x’/line y’	I	……	

The analysis consisted in describing the different subthemes mentioned, their importance for the physicians (themes mentioned most frequently or at least repeatedly during an interview, those appearing spontaneously, and so on), their consensual nature (or not), and the relations between them.

### Ethical approval

We did not submit our study to an ethics committee because this approval is not required for qualitative research studies in France. But we rigorously applied the standard ethical requirements for such studies: we requested the written consent of participants (all of them general practitioners) after explaining the study’s purpose and procedures, including the anonymization of every aspect of the transcripts that could enable identification of the participants and their right to withdraw from the study at any point.

## Results

In reporting the results, we state that a theme was mentioned spontaneously or in response to a question only when the type of mention was similar for most of the doctors we interviewed.

### Description of the sample

Of the 50 physicians we reached, 32 agreed to participate and were finally interviewed (64%). Nineteen (59%) were men and 21 (66%) were aged 50 years or older. The proportions in group and solo practices were identical. They had been practicing medicine for a mean of 24 years (range: 2–35 years). Nearly half (47%) reported participating in a continuing medical education program on mental health during the previous 3 years. Of the 15 GPs who declined to participate, 47% were men and 40% 50 years or older.

### Opinions about psychotherapy

GPs rarely mentioned psychotherapy spontaneously. Participants defined psychotherapy as supportive interviews conducted by a professional with specific training, in contrast to the informal support that they provide. They think that psychotherapy enables patients to confide in someone, to unload or unburden themselves emotionally: a psychotherapist helps patients to work on themselves, to identify the cause of their ill-being, and to mobilize their personal resources.

The GPs defined the informal support that they provide to patients in the same terms, but recognized that they have neither the theoretical framework nor the academic training to provide psychotherapy.

Their discourse on the subject of psychotherapy was unspecific and mentioned only a few themes: its utility and relevance for patients with depression, its dependence on patient adherence, and the obstacles to access to it. The different types of psychotherapy, their indications, effectiveness, and benefits were little discussed. Some GPs indicated their preference for cognitive-behavioral therapy; others spoke, more frequently, about psychoanalysis:

“*Lying on a couch and describing your troubles (GP29)*."

“*Often you can only make progress toward understanding the cause of problems by searching, by exploring the repression of memories in the unconscious. Pathogenic experiences are often involved (GP2)*."

We identified three profiles of physicians according to their opinions about treatments for depression and their therapeutic strategies (Table 3). The first profile comprised GPs with very decided opinions, unfavorable to psychotherapy, and with a preference for pharmacological treatment (6/32). The second profile corresponded to GPs with very positive opinions about psychotherapy and notably prudent about antidepressants. These physicians reported that they very frequently suggest psychotherapy to their patients (6/32). The third profile covered GPs with globally positive opinions about psychotherapy but who nonetheless reported reservations about it. The latter applied mixed practices and adapted their prescriptions to each patient.

**Table 3:** Profiles of physicians as a function of their practices and opinions related to psychotherapy and antidepressants.

	Profile 1 (N = 6) Pro-medication physicians	Profile 2 (N = 6) Pro-psychotherapy physicians	Profile 3 (N = 20) Physicians with mixed practices
**Management practices**	Frequent prescription of antidepressants Few/no suggestions of psychotherapy Initiation of management, often early	Prudence about medication Prescription of antidepressants as a last resort Psychotherapy proposed almost routinely	Variable practices: Possible prescription of antidepressants and/or psychotherapy, depending on the situation Consideration of patient's preferences
**Opinions about antidepressants**	Very effective, sometimes miraculous Well tolerated Little mention of risks No reason to limit the antidepressant prescriptions	To use only in the most severe cases, but sometimes essential Uncomfortable prescribing antidepressants	Sometimes indispensable Medication makes it possible to manage acute situations, but does not cure Must not be prescribed whenever or however
**Opinions about psychotherapy**	Slightly if at all effective or useful Not a real treatment, but rather a personal process	A treatment in its own right of depression Effective Desirable for most patients with depression	Treatment with advantages and disadvantages Variable effectiveness, substantial obstacles to access Make it possible to act on the cause of the disorder Cannot be offered systematically to all patients
**Physicians' characteristics**	Men Older physicians	Women Younger physicians	No associated characteristics
**Examples of verbatim statements**	*"Well*, *I admit I'm not very 'psychotherapy' (…) as if the shrink is a magician able to erase problems; for me*, *that's not medicine*.*"*	*"If it was just my choice*, *all my depressed patients would be in psychotherapy (…) I consider it’s the key to recovery*.*"*	*"It can work*, *but not all the time"*. *"[The patients] must be capable of reflection*, *of analysis (…)*, *have some minimum level of education (…)*. *It's not accessible to everyone*.*"*

### GPs' collaboration with mental health professionals

Most of the GPs spontaneously mentioned their relationships with mental health professionals, mainly psychiatrists. Participants mentioned the following themes (Table 4): the frequency of, reasons for, and obstacles to referring patients, GPs' relationships with these mental health specialists, and the differences in practices between GPs and psychiatrists.

**Table 4:** Themes raised by physicians on the subject of their collaboration with mental health professionals.

Themes	Subthemes	Occurrences
**Orientation of patients in a depressive episode**	**Frequency of referral of patients with depression to a professional specialized in mental health**	12/32
	- Refer rarely	7/12
	- Never refer	3/12
	- Always refer	2/12
	**Patients whom the GP does refer to a specialized professional**	22/22
	- Severe disorder	12/32
	- Case that is a problem for the physician	5/22
	- Suicide risk, suicidal ideation	4/22
	- Failure of the treatment by the GP	3/22
	- The patient needs to talk to someone else	3/22
	- Chronic nature of disorder	2/22
	- Patient experienced a trauma	2/22
	- Psychiatric comorbidity	1/22
	- Requires the prescription of medication	1/22
	- Disagreement between the physician and the patient	1/22
	**Criteria for choosing a specialist**	17/32
	- Leave the choice to the patient	3/32
	- Recommend a professional:	14/32
	• with whom the GP has a good relationship/works with regularly	11/14
	• who practices near the patient's home	4/14
	• who spends time with patients	3/14
	• about whom the GP has had good feedback from patients	2/14
	- Write a referral letter to the specialist	5/32
	- Refer differently according to the patient's needs	2/32
	**Difficulties related to the referral encountered by the physicians**	23/32
	- Difficulty of obtaining an emergency appointment	12/23
	- Refusal of some patients to consult a specialist	12/23
	- Cost for the patients	10/23
	- Time to the first appointment too long	9/23
	- Don't know to whom they should refer the patient	7/23
	- Insufficient supply of specialists	3/23
**GPs' perception of their role and that of private-practice psychiatrists in managing depression**	**GPs' perception of their role and that of private-practice psychiatrists in managing depression**	20/32
	- GPs manage all mental health disorders, except for the most severe cases	12/20
	- GPs manage patients with neuroses, psychiatrists those with psychoses	6/20
	- The role of the GP is to screen and refer patients with mental health disorders	4/20
	- GPs can replace a psychiatrist when the patient refuses to consult a specialist	3/20
**Differences in practices between GPs and psychiatrists**	- The GP is closer to the patient, knows him/her better than the psychiatrist	5/32
	- The psychiatrist is an expert in mental illness, the GP is a clinician	6/32
**Opinions of general practitioners about their own practices**	- Most often, the GPs have no difficulty managing patients with depression	16/32
	- The GPs feel that they manage depression effectively	12/32
	- They are used to facing mental health problems and have acquired experience with these issues	9/32
	- They do not have adequate training in psychiatry	7/32
**Relation with specialists**	- Communication between general practitioners and psychiatrists is inadequate	10/32
	- Psychiatrist do not respond to GPs, even when the latter have written to them	4/32

Of the 12 participants who mentioned the frequency with which they referred their patients to mental health specialists, 10 said they did so only in complex or severe situations.

Besides the difficulties in access to psychiatrists (long wait time for appointments, cost of consultations with specialists in private practice) mentioned by most (23/32), more than half the GPs also reported that they were dissatisfied with their current collaborations (19/32). They complained especially about a lack of communication (10/32): the rarity of conversations about the patients, the specialists’ failure to respond to GPs about the referral, and mutual difficulties in understanding one another. Some GPs reported experiencing more problems working with psychiatrists than with other specialists.

"*The dark spot is the (…) lack of communication with psychiatrists (…) we all communicate with the other [specialists] except them, it's strange (GP26)*."

Nonetheless, the majority of GPs wanted to communicate more with psychiatrists and coordinate better with them, to improve the quality of care, to facilitate the patients' care plan, and to break their own isolation.

"*There is perhaps not enough dialogue between general practitioners and private-practice psychiatrists. Nonetheless, I would really like for us to succeed in talking more often and better. I think it would be good, it could only be useful for everyone (GP3)*."

### GPs' perception of their own and psychiatrists' roles and practices

#### Management of depression

Nineteen participants mentioned the respective roles of GPs and psychiatrists in managing patients with depression. Twelve thought that they should manage depression while psychiatrists should care for psychotic disorders and patients at risk of suicide. Some GPs considered that they had acquired the knowledge and skills necessary for managing depression through experience, despite their lack of initial training in mental health. Psychiatrists nonetheless remain a potential resource should they encounter difficulties in caring for a patient (11/19).

"*Depressed patients, we see them every day or almost, we're used to them, we recognize them, we know how to handle them, to treat them. (…) and then if really we are having difficulty, if we see that truly the patient is not doing well, that things are dragging, we can always contact a psychiatrist at that point (GP4)*."

"*Finally, we can manage all mental health disorders on an outpatient basis, except for crises obviously (GP10)*."

Other GPs (5/19) had a different vision and reckoned that everything linked to mental health should be handled by psychiatrists, whom they perceived as experts with more relevant skills than GPs. Among these 5 GPs, none belonged to the first profile very favorable to pharmacological treatment.

"*We are clearly not in the same league…it's their specialty…everything that is a hard case, in quotes, mental illness, that's their domain (…) there is a reason that it's a specialty in its own right, and there's a reason we call them when we can't manage with some patients (GP6)*."

Some GPs considered that their role is principally to identify patients with depression and refer them to specialists.

"*We are better equipped to identify diseases than psychiatrists. That's the heart of our work: screening, recognizing, and directing to specialists. Well, it's the same for mental health (GP26)*."

#### GPs' common vision of depression management

Despite these divergent views of their role, a common vision of appropriate management emerged from the GPs' discourse: the patient is at the center of the care, which must be comprehensive and adapted on a case-by-case basis, according to his or her characteristics, personal life story, and environment.

"*It's the core of our work (…): we are constantly obliged to adapt to each of our patients, to deal with their history, their family, their situation. Each patient is unique, each decision we make must also be unique (GP23)*."

Most of participants stressed their relational skills: listening, supporting, and advising are the heart of their work, an integral part of the management of depression, and what patients expect of their GPs.

"*Listening is the basis of my work, especially in diseases like that. It's true that I take an enormous amount of time with patients, that I sometimes find myself with patients who spend nearly 45 minutes in the office. So yes when I'm running late, they complain a little, but otherwise, they appreciate being listened to and that’s what they come to see me for (GP28)*."

#### A language close to that of their patients

Vocabulary used by GPs to designate depression was mostly non-technical, very close to ordinary daily vocabulary. Only one GP mentioned DSM IV, and two used the term "depressive episodes.” Instead they mainly used terms such as "true depression," "small/big depression," "deep or serious depression," neurotic/nervous disease," and "very depressed.” Similarly, some physicians seemed to distance themselves from the medical terms usually used in psychiatry, even as they used them.

"*It’s up to us to distinguish between a true depression, a major depressive episode (…) as we are supposed to call it (GP2)*.”

"*We talk about depression as it's written in books, with abulia, apathy, disinterest, the person doesn’t even comb their hair anymore, doesn't eat, and blows up over nothing, starts to blubber, and that, those are depressive symptoms (GP21)*.”

#### A comparison of their practices with those of psychiatrists

Participants frequently compared their practices to those of psychiatrists (Table 5). They described themselves as closer to their patients, listening to them better than the psychiatrists did. Some reported negative feedback from patients describing psychiatric consultations that were too short (11/32) and psychiatrists who did not listen (8/32). They described their practices as less technical than those of psychiatrists, based on pragmatic field experience, contrary to psychiatrists, with their scientific expertise.

**Table 5:** Principal perceived differences between the practices of GPs and psychiatrists.

	General practitioners ("us")	Psychiatrists ("them")	Verbatim excerpts from interviews
**Relationships with patients**	Close to patients Know their history and their environment Empathetic, listening Available, spend time with patients	Distant and cold with patients Know little about their patients and their histories One-off consultations	*“We know the families (…) I know their environment*, *I know their husband*, *I know their children (…) obviously*, *the first time the psychiatrist sees you*, *he doesn't know your children or your husband“*.
**Practice**	Clinical, intuitive Comprehensive individualized (case-by-case) management Routine clinical questioning Vocabulary close to that the patients use	Technique, position as expert Use of screening tools Reference to the literature and to diagnostic classifications Scientific vocabulary	*"I'm a clinician (…) I don't like this kind of tool [diagnostic scales] and then I base my judgments on the symptoms I observe; I'm a clinician*. *I leave that to the psychiatrists"*.
**Expertise**	Pragmatic Based on professional experience	Scientific Education and training	

## Discussion

### Principal results

Our qualitative study is the first in France to analyze in detail the opinions of GPs about psychotherapy, their relationships with mental health professionals, and their perceptions of their profession, their role, and those of psychiatrists, and to describe the relations of these factors with GPs' strategies for managing major depression.

One of its principal results is that GPs had distinct attitudes toward psychotherapy, falling into three different categories according to their opinions of this type of treatment (Table 3). The majority of GPs were reasonably favorable to psychotherapy. They perceived its usefulness but also underlined its disadvantages. Some GPs were very favorable to psychotherapy, proposing it almost routinely to patients with depression. On the contrary, some GPs opposed to this type of treatment. These opinions were consistent with their treatment choices for patients with depression.

Their collaboration with psychiatrists was a major concern of the GPs: they considered their relationships with these specialists unsatisfactory and asked for more and better collaboration with them.

Finally, the participants shared the same concept of management in general practice—comprehensive and individualized patient management, relational skills, pragmatic knowledge based on experience; and they distinguished these practices and methods from those they attribute to psychiatrists.

### Strengths and limitations of the study

Several strengths associated with the methods used in the study should be underlined. The GPs were selected by a random drawing that was stratified for age and sex to ensure the participation of GPs of different ages and sexes, because these variables are often associated with differences in physicians’ care practices [6,15,16]. Our study was thus able to examine the diversity of GP’s opinions and points of view in terms of their demographic characteristics. Next, two researchers collected and analyzed the data. The resulting triangulation strengthened the validity and reliability of the data produced [17,18]. This survey also has some limitations. First, it is possible that physicians who agreed to participate in this survey are more interested in or more frequently faced in their practice by depressive disorders than the non-participants; as compensation, however, it increased the wealth of points of view about the different ways of managing them. In view of the size of the sample, prudence is necessary in generalizing these results. Nonetheless, interviewing 32 GPs allowed us to meet a wide variety of GPs and to attain theme saturation. Moreover, the similarity of some of our results to those in a quantitative study of a representative national study of French GPs [7] suggests that they are not specific to the GP population we interviewed. The second limitation stems from its entirely urban setting. Because the supply of specialists in mental health in rural areas is considerably smaller than in cities, the inclusion of rural GPs might have modified our findings about GPs’ use of psychiatric referrals and their modes of collaboration with psychiatrists.

### Interpretation of results

#### Differing opinions of psychotherapy

The first objective of this survey was to improve our understanding of the paradox that general practitioners, despite their favorable opinions of psychotherapy, rarely suggest it to patients with depression [6]. Our results, which show that GPs differ in their opinions of psychotherapy, qualify the preceding observation. Ardent defenders of psychotherapy accounted for only a minority of the GPs in our sample. The others, less favorable to psychotherapy, tended to offer this treatment less often to their patients, which may help to explain the low rate at which GPs in France refer their patients with depression for psychotherapy [1].

GPs' discourse about psychotherapy was sparsely furnished and referred principally to psychoanalysis: long and expensive treatment, Freudian theories of the unconscious, etc. Other types of psychotherapy were mentioned very little. This is in line with other study results showing that GPs express themselves about psychotherapy and especially psychoanalytic approaches in ways that match the perceptions of laypeople [19]. This suggests that GPs may share with laypeople representations about psychotherapy and psychoanalysis.

Those results might reflect GPs' lack of training in and knowledge of psychotherapy: previous results show that most GPs (82%) would like to be better trained about psychotherapy [6].

The GPs' relatively infrequent mention of psychotherapy and the few themes related to them may also be related to some of their attitudes toward therapy: some GPs do not consider it to be a treatment for depression to the same degree as drugs are, but rather an adjunct treatment. In a qualitative survey in Sweden, every GP questioned responded that psychotherapy could not replace pharmacotherapy in patients with major depression [13].

More generally, the pharmacological model for the management of depression is dominant among GPs [13,20–23], and antidepressants remain the strategy most frequently used by GPs in the treatment of depression, even mild to moderate, in France [6], as abroad [20,23]. Most physicians in France have a positivist view of drugs, are persuaded that continuous progress occurs in their development, and moreover tend to underestimate the risks and side effects of the treatments they prescribe [24]. They are also notable for their particularly high level of medication prescription: 90% of GP visits in France conclude with a prescription, compared with 72% in Germany and 43% in the Netherlands [25].

#### Difficulties of collaboration between GPs and psychiatrists

As in our quantitative national survey of GPs [6], the participants in this study explained their low recourse to psychotherapy by the existence of obstacles to access to this type of treatment, linked to what they consider to be both its insufficient supply and its high cost. But the results of this qualitative study also suggest that the difficult collaboration between GPs and psychiatrists is another major obstacle to the referral of patients with depression.

Numerous publications in various countries have pointed out the difficulties of collaboration between GPs and mental health professionals, as well as GPs’ dissatisfaction with it [9–11,13,26–31]. Like ours, these studies show relational difficulties between GPs and psychiatrists, including lack of communication and difficulties in understanding each other. Nonetheless, the reasons for these relational difficulties merit examination. Most GPs expect positive effects from this collaboration, in terms of continuity and quality of care and access to it, and would like to strengthen it [9,10,13,27,29,32].

Previous publications have already reported that GPs, like some of those participating in this study, report that they find it harder to work with psychiatrists than with other specialists [11,29,33]. One hypothesis that might explain this is the lack of a clear definition of the respective roles of GPs and psychiatrists in the organization of care and follow-up for depression, at least in France [34]. In a study of Belgian GPs and psychiatrists in 2009 [35], more than half the psychiatrists thought that it was preferable for patients who need antidepressant treatment to be managed by a psychiatrist, while only 3% of GPs agreed. Similarly, 74% of the psychiatrists, but only 47% of GPs thought it was better for specialists to conduct the psychotherapy of patients with depression. These results show that GPs and psychiatrists have different visions of the management of depression and of their respective roles in it. This context may enhance the perception of competition between them in this particular context of patients with depression [36], at least for GPs and psychiatrists in private practices.

#### GPs' strong professional identity

Several elements testify to the existence of a strong common professional identity among GPs, based both on the values and principles of practice shared by GPs and on their strong differentiation of their management practices from those of psychiatrists.

GPs stressed their relational skills and their experience in the field more than their technical and medical skills. This suggests their willingness to be close to their patients and to satisfy their needs, especially one of their principal needs—to be heard and listened to [37]. It also points out GP’s insistence on affirming the specificity and added value of general medicine compared with other specialties.

The distancing from psychiatrists goes hand in hand with the affirmation of GPs' skills and a devaluing of the same skills in psychiatrists, but without devaluing the latter's technical competence. According to the theory of social identity developed by Tajfel and Turner [38], who proposed a framework for studying intergroup conflicts, individuals belonging to the same professional group can tend to accentuate the resemblance between the members of their own group and their differences compared to members of other groups, which thus leads to discrimination against the others. This process would allow GPs to maintain their profession’s positive social identity, although they may also have a devalued perception of their function relative to specialists [39]. It also makes it possible to reaffirm their legitimacy and competence in the management of depression, in a context in which their drug prescription practices are the object of substantial social criticism [40–42].

This professional identity may present an obstacle to the collaboration of GPs and psychiatrists. Shared values, the existence of a common language, and the mutual impression that collaboration will improve the quality of care are essential prerequisites for the development of an interprofessional collaboration [10]. Improving the collaboration between GPs and psychiatrists thus appears to require the development of a common culture between them with a shared vision for the management of depression and of the role each plays for the patient.

#### Conclusions

The results of this study provide new avenues for explaining the low rate of referrals by GPs for psychotherapy in France. They show the interest of taking into account GP’s opinions about psychotherapy and about mental health professionals, as well as their perceptions about their profession and their role in the management of depression. Several types of activities aimed at improving the cooperation between general practitioners and mental health professionals could be tested and assessed in France [10,34]. They include systems of collaborative care, the effectiveness of which have been demonstrated on several occasions [32], and the development of interdisciplinary training [31], common to GPs and psychiatrists practicing in the same area. These types of activities should promote the development of a common culture between these professionals and help to create local informal care networks across the country.

## Supporting information

S1 Appendix Interview guide. (DOC)

S2 Appendix Information sheet to participants. (DOC)
