# Supplementary material for: Opinions of general practitioners about psychotherapy and their relationships with mental health professionals in the management of major depression: A qualitative survey
Source: PLoS One. 2018 Jan 31;13(1):e0190565. doi: 10.1371/journal.pone.0190565 (PMC5791973; doi:10.1371/journal.pone.0190565)
Supplement: S1 Appendix — (DOC) [file pone.0190565.s001.doc]

Marseille, April 7, 2011

Dear Colleague,

INSERM and the Regional Health Observatory for Provence-Alpes-Côte d’Azur (ORS PACA) are conducting a study among physicians in private practice about the management of mental health disorders. The study was initiated and is co-led with a group of both private practitioners and hospital staff physicians. Its objective is to gain a better understanding of the importance of this issue in their daily practice and to examine the difficulties they face.

The study is based on qualitative interviews with general practitioners, which will last around 20 minutes. These interviews are strictly anonymous and confidential. You will receive as compensation the cost of two patient consultations. An investigator for ORS PACA will contact you soon, to obtain your consent to participate in this survey. We thank you for welcoming him/her.

Because your experience and your point of view are essential to us, we count strongly on your participation. We remain at your disposition for any additional information. With our collegial greetings,

Dr Pierre Verger, Directeur of Research, ORS PACA


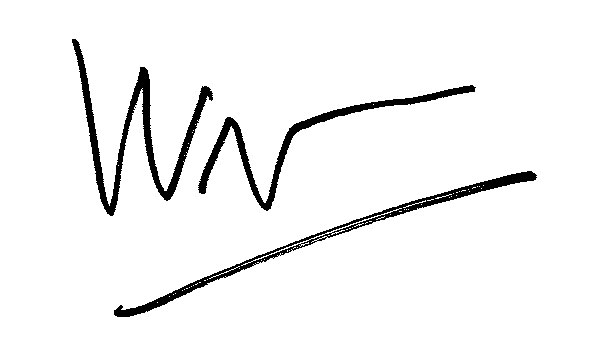


Dr Hervé Pegliasco, President of the national association for the coordination of continuing education and evaluation in specialized medicine (A.FOR.Spé)

Contacts :

Jean-Marc Manzi, 04.91.59.89.12

Hélène Dumesnil, 04.91.59.89.23
